# Multiplexed immunofluorescence analysis of CAF-markers, EZH2 and FOXM1 in gastric tissue: associations with clinicopathological parameters and clinical outcomes

**DOI:** 10.1186/s12885-022-10312-0

**Published:** 2022-11-18

**Authors:** Hui Sun, Xin Wang, Xiaoyan Zhang, Xu Wang, Cong Tan, Weiwei Weng, Meng Zhang, Shujuan Ni, Lei Wang, Dan Huang, Midie Xu, Weiqi Sheng

**Affiliations:** 1 Department of Pathology, Fudan University Shanghai Cancer Center, 270 Dong’an Road, Shanghai, 200032 China; 2 Department of Oncology, Shanghai Medical College, Fudan University, Shanghai, 200032 China; 3 Institute of Pathology, Fudan University, Shanghai, 200032 China

**Keywords:** EZH2, FOXM1, Cancer associated fibroblast, FAP, Prognosis, Gastric cancer, Immunohistochemistry

## Abstract

**Background:**

The aim of this study is to explore the expression and clinical relevance of CAF-associated markers, EZH2 and FOXM1 in gastric samples.

**Methods:**

Protein expression were detected and evaluated by multi-plex immunofluorescence (mIF) in 93 cases of gastric cancer (GC) and 31 cases of gastric intraepithelial neoplasia (GIN). The correlation among their expression, and the relationship of them with clinicopathological parameters and prognosis in GC were then analyzed.

**Results:**

FAP was specific expressed in the CAFs of GC samples, and thus be utilized as a CAF-associated marker in our subsequently analysis. The immunostaining of EZH2, FOXM1 and FAP were significantly upregulated in patients with GC tissues than in those normal gastric mucosa or GIN tissues. The average fluorescence intensity of FAP was slightly positively correlated with EZH2 in GC, GIN and normal samples, whereas the percentage of FAP positive cells has no correlation with that of EZH2. Both the percentage of positive cells and the average fluorescence intensity of FOXM1 were positively correlated with that of FAP and EZH2 in GC, GIN and normal samples, except for FOXM1 and EZH2 expression in normal tissue samples. No significant association was observed between FAP expression and any clinicopathological parameters, whereas the positive frequency of EZH2 and FOXM1 were correlated with tumor location significantly and tumor invasion depth, respectively. In addition, there was strong positive correlations between FAP protein expression and overall survival (OS) and disease-free survival (DFS), and EZH2 expression was positively associated with OS in patients with GC. Furthermore, EZH2 and FAP protein expression was an independent prognostic factor for OS and DFS, respectively.

**Conclusions:**

These results suggest that both EZH2 and FOXM1 expression was positively associated with CAFs abundance in GC. They may be potential cellular target for therapeutic intervention, especially in patients with a large number of CAFs.

## Background

Gastric cancer (GC) is one of the most common cancers, although, with a decreasing incidence worldwide, it is still one of the leading causes of death in China [1, 2]. Although treatment regimens for advanced GC involve combination surgery and chemoradiotherapies, it is still difficult to increase long-term surviva l[3]. In present, no regimen has become a globally accepted standard, so there is urgency needed for new therapeutic options for the clinical prognosis and treatment of GC. The limited benefit of treatments for GC is increasingly attributed to the tumor microenvironment (TME), including stromal cells, cancer stem cells, cancer cells, immune cells, pericytes, endothelial cells (ECs) and cancer-associated fibroblasts (CAFs), as it is well known that the profuse tumor stroma in TME contributes to GC progression [4]. In addition, immunotherapy and molecular therapy have received unprecedented attention and became additional method of GC treatment due to its remarkable curative effect and low side effect s[5, 6]. However, none of immune and molecular biomarkers showed satisfactory specificity and sensitivity. Hence, to discover specific prognostic biomarkers and novel promising therapeutic targets for GC, the role of tumor stroma cells in the development and progression of GC should be explored more intensively.

Cancer-associated fibroblasts (CAFs), a key source of the ECM that contributes to the desmoplastic stroma, are the most abundant stroma cells in the GC TME, and play crucial roles during cancer malignant progression [7]. The identification of CAFs is usually based on the expression of various “CAF markers”, including but not limited to: platelet-derived growth factor receptors (PDGFR), vimentin, α-smooth muscle actin (α-SMA), fibroblast-specific protein 1 (FSP1, also known as S100A4), and fibroblast activation protein alpha (FAP), which separates them from the larger pool of fibroblasts present in the body [8].α-SMA is not only used to identify CAFs with a myofibroblast phenotype, but also used as a general marker for vascular muscular cells and pericytes [9]. FSP1, is not only used to identify fibroblasts, but also used as marker for epithelial cells, macrophages and other immune cells [9, 10]. It is reported that FSP1 may serve as quiescent or resting fibroblast marker [9]. FAP, as a more commonly known marker of activated CAFs, as a more commonly known marker of activated CAFs, is upregulated in the numerous of epithelial tumor stroma [11]. This has contributed to the widespread use of FAP as an identifier of potential CAF populations, and which might be one of the most promising therapeutic targets of CAFs.

The enhancer of zeste homologue 2 (EZH2) belongs to the family of polycomb group genes (PcGs), that can alter gene expression by trimethylation of histone 3 lysine 27 (H3K27me3) [12]. It has emerged as a master regulator of intracellular physiological and pathological processes [12]. Cumulative evidences shows that EZH2 is overexpress in many cancer types including breast cancer, esophageal cancer, and GC [13–15]. We also have reported that EZH2 can promote tumor cell growth, metastasis and stemness in GC [16]. As a transcriptional coactivator, EZH2 can interact with transcription factors and thus implicated in the expression of downstream target genes. Recently, Mahara, et al. demonstrated that EZH2 can promote cancer invasion by form complex with forkhead box M1 (FOXM1) [17], which is a classic proliferation-associated transcription factor that involves in almost all hallmarks of tumor cells [18]. Interestingly, recently CAFs-enhanced EZH2 expression has been disclosed and is associated with migration ability of tumor [19]. And FOXM1 was reported mediates CAFs’ tumor-promoting property in hepatocellular carcinoma [20]. However, the relationship between the expression of EZH2 and FOXM1 in GC tissue, and the correlation between their expression and CAFs in the GC microenvironment, has not been fully investigated.

In the present study, we aim to analyze the expression of EZH2, FOXM1 and CAFs markers (α-SMA, FSP1 and FAP) in GC tissue by using mIF, and tried to analyze their correlation, the correlation between FOXM1 or EZH2 positive GC cells with CAFs, and their correlation with clinicopathological parameters and prognosis of GC.

## Materials and methods

### Patients and samples

A series of 10 × 12 tissue microarray (TMA) that included 100 cases of GC and normal gastric tissue samples, and 34 cases of GIN tissue samples, which was also used in our previous reports [21, 22]. The TMA were obtained from Fudan University Shanghai Cancer Center (FUSCC) Tissue Bank. Each case had one repeat core to preclude loss of samples. A total of 93 tumor and adjacent normal tissue samples, and 31 GIN samples were interpretable in the TMA analysis. The remaining 10 samples were not analyzable due to the lack of unequivocal tumor cells or loss of the tissue spot during the technical procedures. The TNM classification system was used to evaluate the GC clinical stage and degree of differentiatio n[23]. None of the patients underwent preoperative treatment, some of the GC patients underwent postoperative chemotherapy.

All specimens were collected from the patients with informed consent, and our study was approved by The Research Ethics Committee of FUSCC.

### Immunohistochemistry (IHC) staining

FFPE sample was selected from the archives of the Pathology Department of FUSCC. Tissue specimens were fixed in formalin and embedded in paraffin and then sliced into sections of 3-μm thick,and and incubated with primary antibodies against FAP (ab207178, abcam,1:250 dilution), FSP1 (ab19896, abcam,1:500 dilution) and α-SMA (D4K9N, Cell Signaling Technology, 1:100 dilution) at 4 °C overnight. The sections were then incubated with a horseradish peroxidase-conjugated secondary antibody (Gene Tech, Shanghai) for 30 min at room temperature. Appropriate positive and negative controls were run with each batch. IHC results were evaluated by three certificated pathologists who were blinded to patient information.

### Multiplex immunofluorescence (mIF)

Assessment of protein expression of CAF-markers, EZH2 and FOXM1 was performed by mIF technology. For mIF staining, CAF-markers, EZH2 and FOXM1 antibody panel and Opal 7-color manual IHC kit (50 slides kit, Perkin Elmer/Akoya, NEL871001KT) was used. Before a run was started tissue TMA slides were baked for 4 hours at 65 °C in an oven. The following primary antibodies were used: FOXM1 antibody (ab207298, abcam,1:200 dilution), EZH2 antibody (D2C9, Cell Signaling Technology, 1:50dilution), FAP antibody (ab207178, abcam,1:200 dilution), FSP1 antibody (ab19896, abcam,1:500 dilution) and α-SMA antibody (D4K9N, Cell Signaling Technology, 1:200dilution). Slides were incubated for 60 minutes with the first antibody and rinsed in three changes of TBST buffer for 2 minute each, followed by detection using Polymer HRP Ms. + Rb for 10 minutes, OPAL dye incubation (1/75 dilution) for 10 minutes. Cycles were repeated for each new antibody to be stained. The nuclei were subsequently visualized by detecting nuclear spectral elements using DAPI. The sections were mounted with ProLongTMDiamond (Introgen™, cat p36970).

### Image acquisition and quantitative analysis

All immunofluorescence-stained slides were scanned using a digital slide scanner (Pannoramic MIDI, 3DHISTECH Ltd), then were independently analyzed and quantified by three experienced pathologists using HALO (v2.2.1870.17, Indica Labs, Albuquerque, NM, USA). Whole slide scan was performed at 100x magnification and multispectral high-power fields were imaged at 200x. To acquire reliable unmixed images, library slides were created by staining a representative sample with each of the specific dyes. This spectral library served as a reference for target quantitation; the intensity of each fluorescent target was extracted from the multispectral data by linear unmixing. As output, the software provides the total number of cell and the positive cell, and the fluorescence intensity of positive cells.

### Statistical analysis

All statistical analyses were performed using SPSS 22.0 (IBM, SPSS, Chicago, IL, USA) and GraphPad Prism version 6.0 (GraphPad Software, San Diego, CA, USA). Comparisons between groups were determined by the χ2-test, Student’s two-tailed t test, and one-way ANOVA. The correlation between the percentage protein positive cells and the fluorescence intensity of positive cells among each marker was assessed by Pearson’s correlation analysis. Survival distribution was compared using Kaplan-Meier methods and the Log-rank test. Univariate and multivariate analysis were fit using a Cox proportional hazards regression model. A two-sided *P*-value < 0.05 was considered significant.

## Results

### Evaluation of CAF markers in GC tissues

**Fig. 1:**
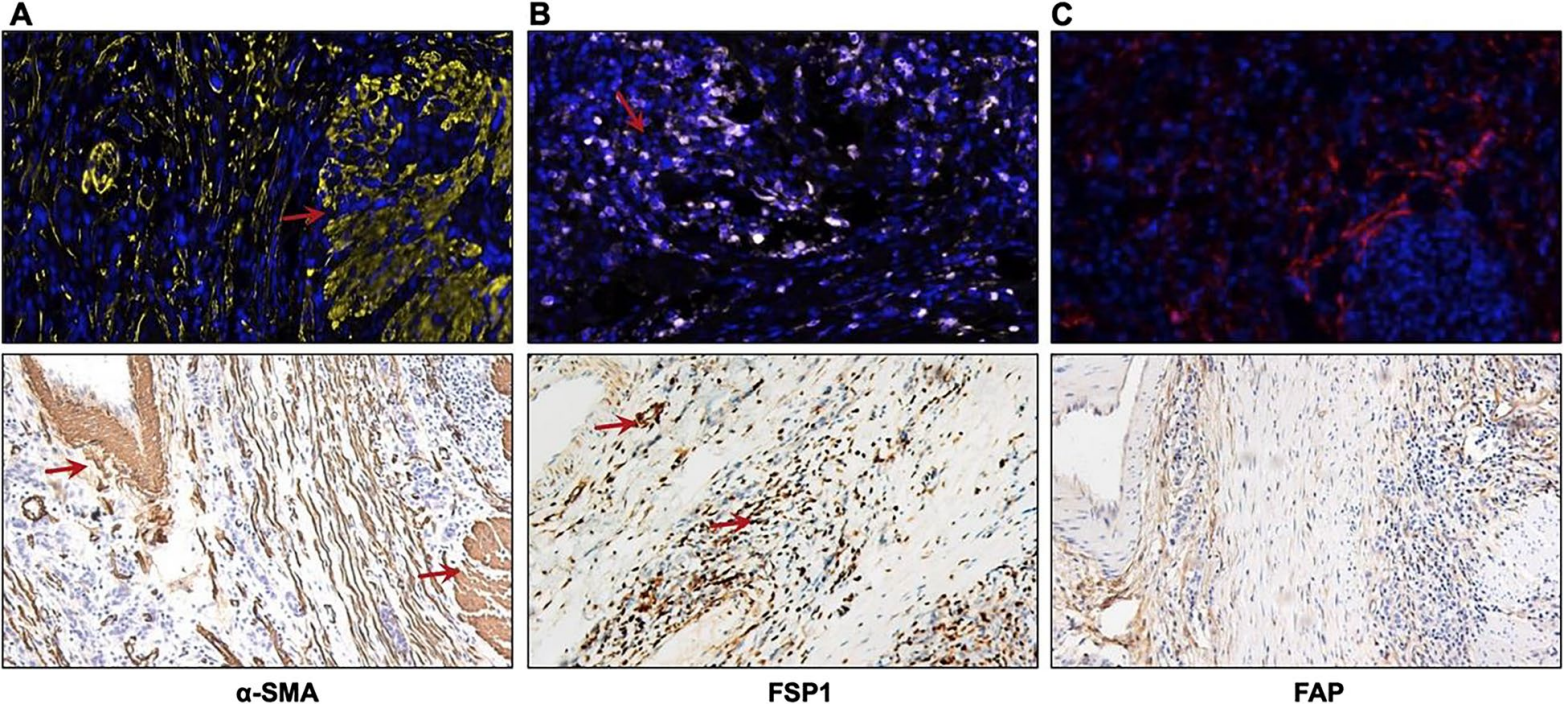
mIF and IHC images showed the distribution of CAF markers in GC. **A** The positive immunofluorescence signals and immunohistochemical results of α-SMA were observed in CAFs with a myofibroblast phenotype, smooth muscle cells (arrows) and pericytes. **B** The positive immunofluorescence signals and immunohistochemical results of FSP1 were observed in CAFs, epithelial cells, and immune cells (arrows). **C** The positive immunofluorescence signals and immunohistochemical results of FAP were observed in fibroblasts

We first evaluated the expression specificity of FAP, FSP1 and α-SMA in GC. Our results showed that in addition to fibroblasts, the positive immunostaining signals of α-SMA were also observed in smooth muscle cells and pericytes of and GC cells (Fig. 1 A); FSP1can also stained myofibroblast, immune cells and epithelial cells (Fig. 1 B). However, FAP is specific expressed in the CAFs (Fig. 1 C). Therefore, FAP is a truly specific marker for CAFs in GC TME, and thus be used as the CAFs marker in our subsequently analysis.

### Expression of EZH2, FOXM1 and FAP protein in gastric tissues

**Fig. 2:**
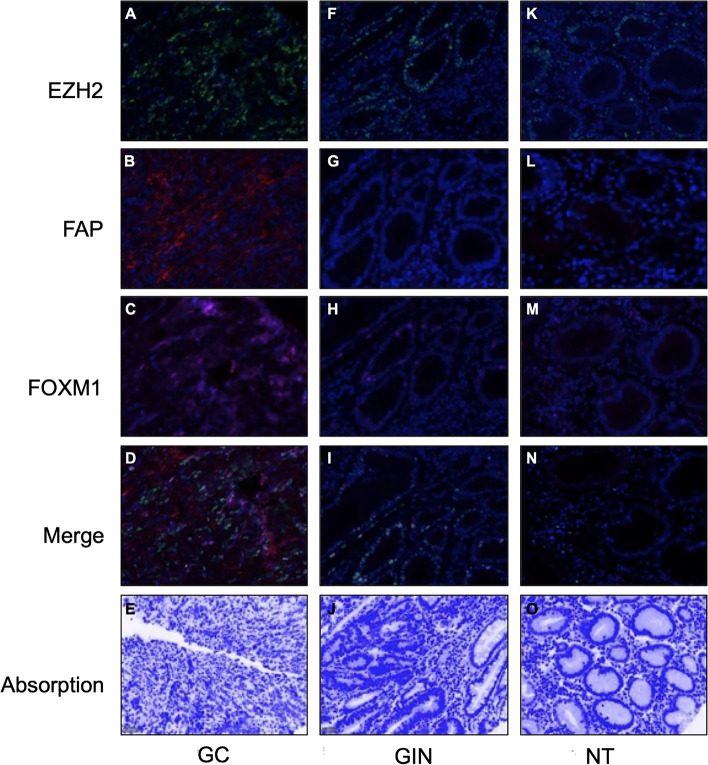
mIF images with individual channels in gastric tissues. The expression of EZH2, FAP, FOXM1, merge and absorption picture in GC (**A**-**E**), GIN (**F**-**J**) and normal (**K**-**O**) tissues. FFPE sections of GC, GIN and normal tissues were stained for EZH2, FOXM1 and FAP, and images were obtained using virtual microscopy Pannoramic scanner (Pannoramic MIDI, 3DHISTECH Ltd)

**Fig. 3:**
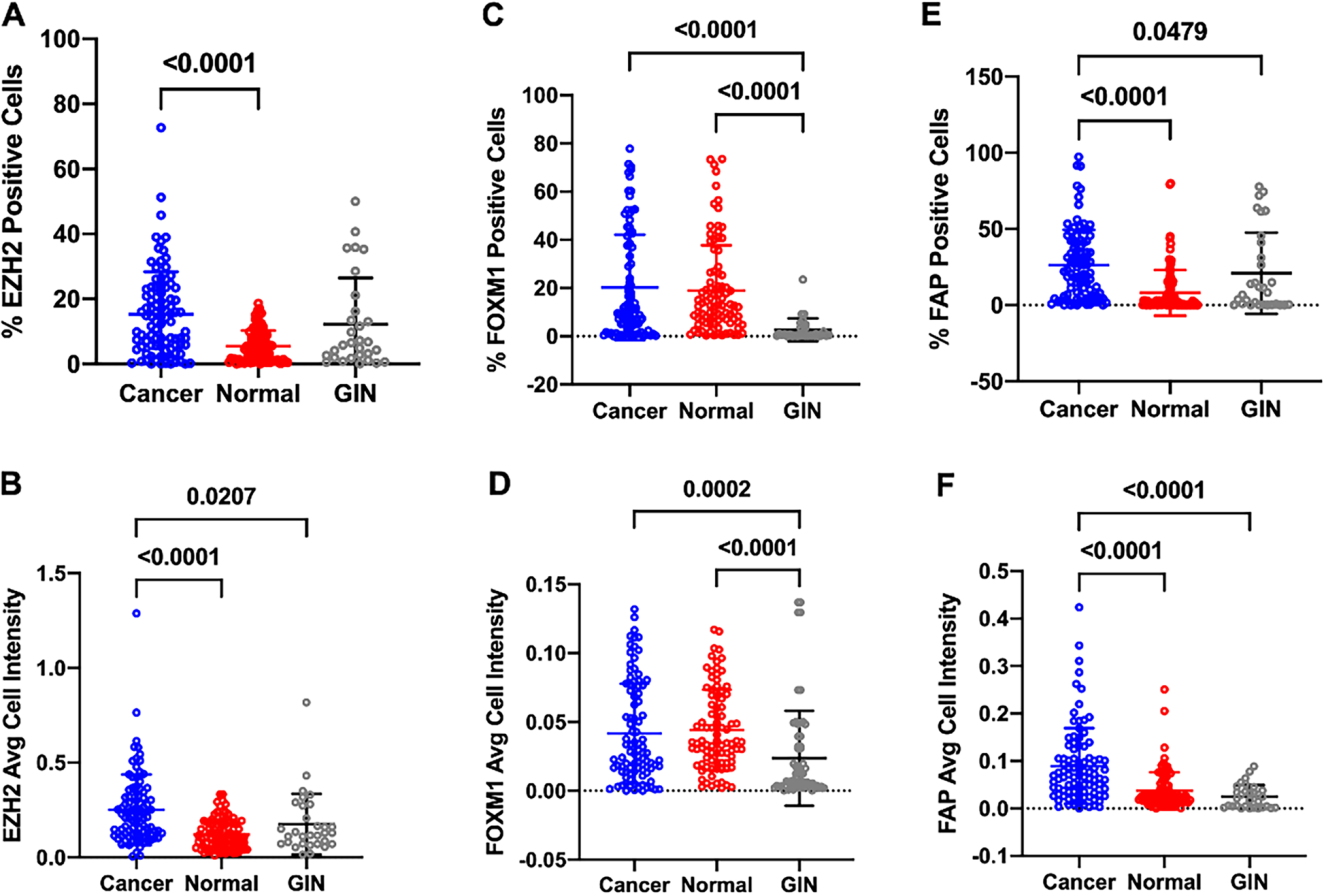
Evaluation of EZH2, FOXM1 and FAP expression in gastric tissue. Scatter plot showed the percentage of EZH2 (**A**), FOXM1 (**C**) and FAP (**E**) positive cell and the average fluorescence intensity of E2H2 (**B**), FOXM1 (**D**) and FAP (**F**) protein in indicated tissue samples. Statistical tests for data among groups were analyzed using one-way ANOVA

The representative mIF result of protein expression in each group of tissue samples is shown in Fig. 2. In comparison with the matched normal gastric samples, both the percentage of positive cells and the average fluorescence intensity of E2H2 protein positive cells were significantly increased in cancer tissues (both *P* < 0.001, with one-way ANOVA, Fig. 3 A-B). However, there was no significant difference between the percentage of positive cells and the average fluorescence intensity of EZH2 positive cells between GIN and normal tissue, yet the percentage of EZH2 positive cell between cancer tissue and GIN (all *P* > 0.05, with one-way ANOVA, respectively, Fig. 3 A-B). For FOXM1 protein expression, although there was no significant difference in its expression between tumor tissue and normal samples (both P > 0.05, with one-way ANOVA, respectively, Fig. 3 C-D); both the percentage of positive cells and the average fluorescence intensity of FOXM1 was increased in cancer tissues than in GIN tissue samples (*P* < 0.001 and *P* = 0.002, with one-way ANOVA, Fig. 3 C-D). Interestingly, the percentage of positive cells and the average fluorescence intensity of FOXM1 in the GIN tissue samples was also reduced compared with the normal samples (both P < 0.001, with one-way ANOVA, Fig. 3 C-D). Compared with GIN and normal tissue samples, both the percentage of positive cells and the average fluorescence intensity of FAP positive cells were significantly increased in cancer tissues (all *P* < 0.05, with one-way ANOVA, Fig. 3 E-F). However, there was no significant difference between GIN and normal samples (both *P* > 0.05, with one-way ANOVA, Fig. 3 E-F).

### Correlation of EZH2, FOXM1 and FAP protein expression in gastric tissues

**Fig. 4:**
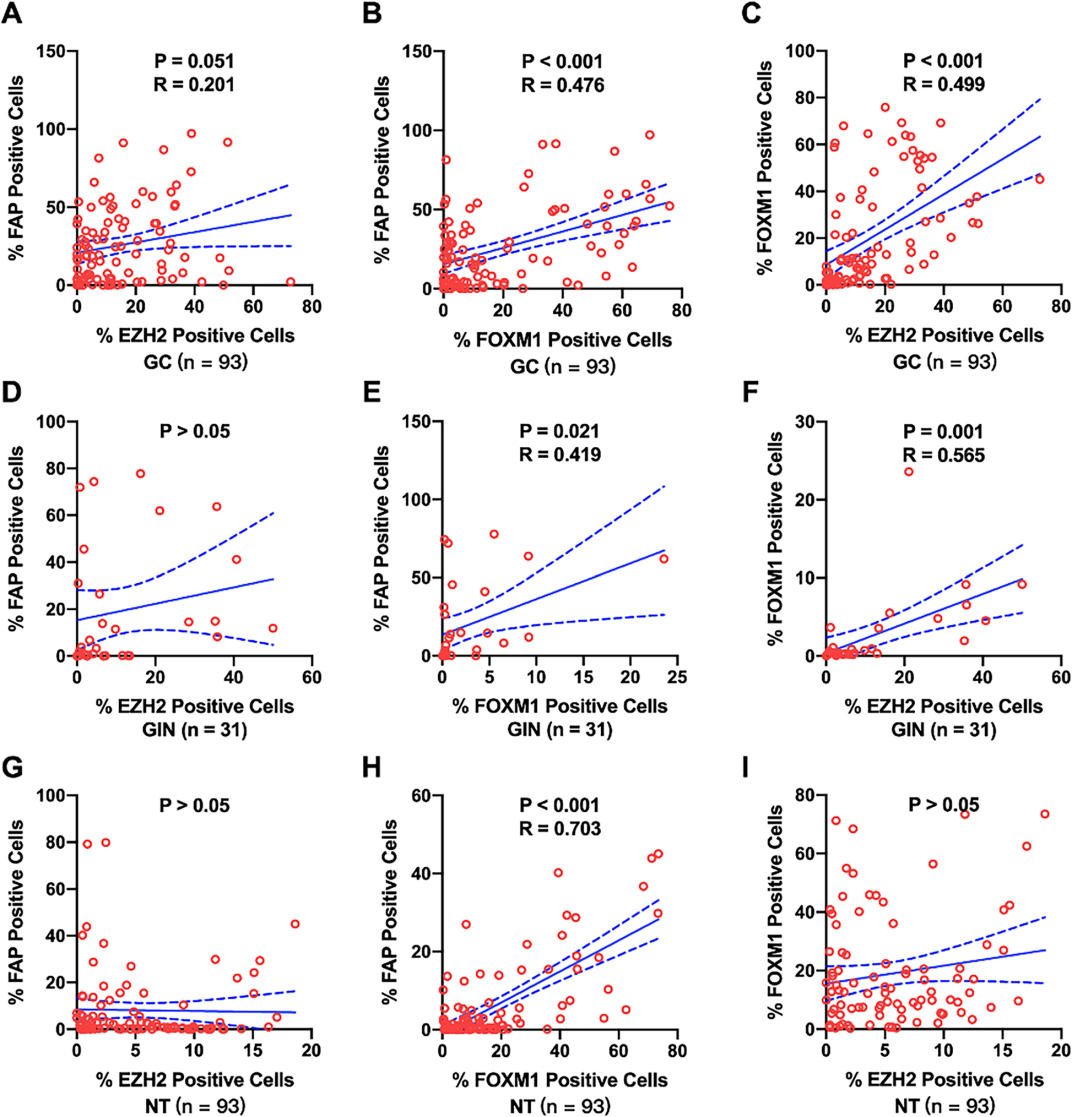
Correlation of the percentage of positive protein cells in gastric tissue. The correlation among the percentage of EZH2, FOXM1 and FAP in GC (**A**-**C**), GIN (**D**-**F**) and normal (**G**-**I**) tissue samples. Statistical tests were analyzed using Pearson’s correlation

**Fig. 5:**
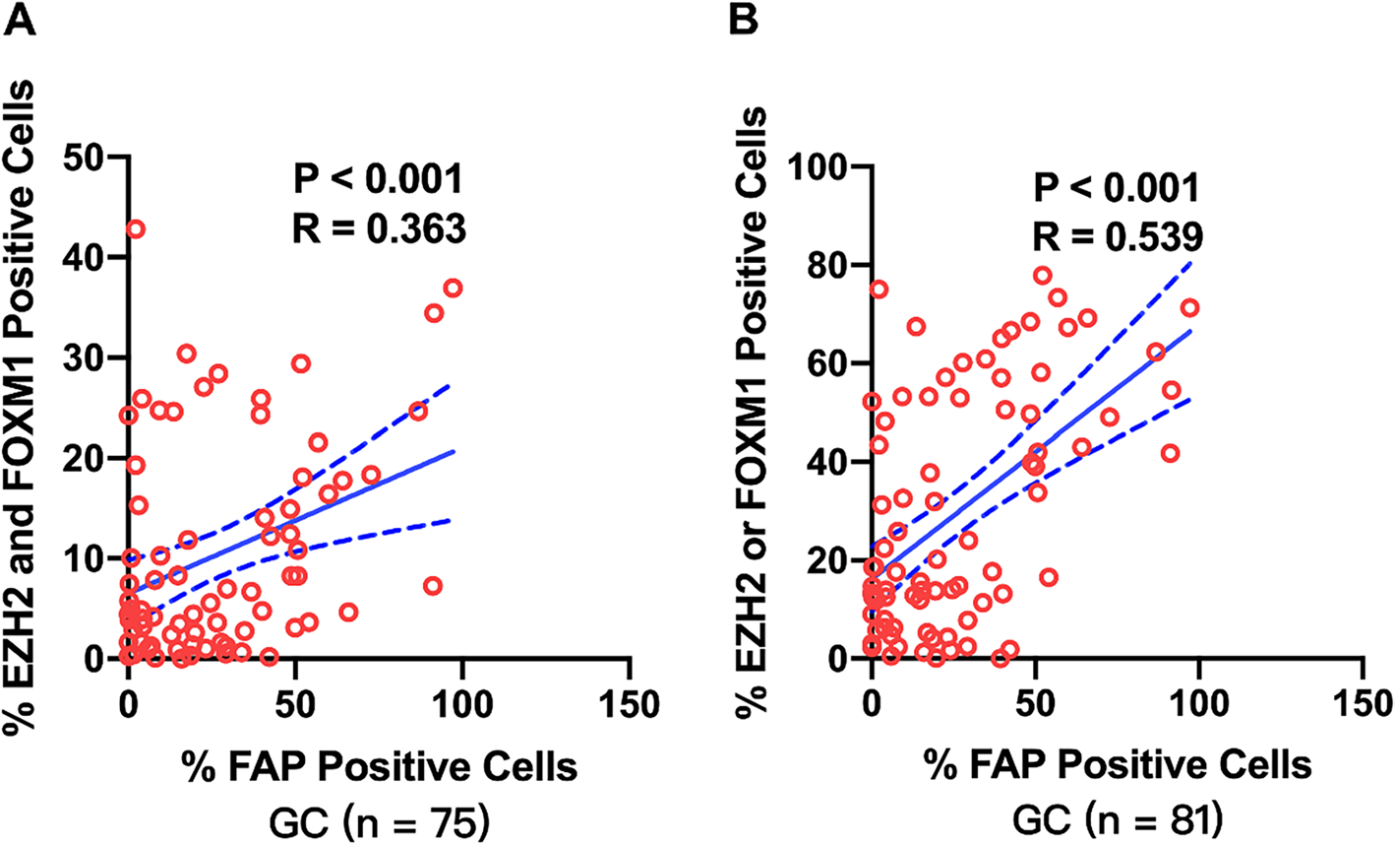
Correlation of the percentage of positive protein cells in GC tissue. **A** The correlation between the percentage of EZH2 and FOXM1 positive cells and those of FAP in GC tissue samples. **B** The correlation between the percentage of EZH2 or FOXM1 positive cells and those of FAP in GC tissue samples. Statistical tests were analyzed using Pearson’s correlation

**Fig. 6:**
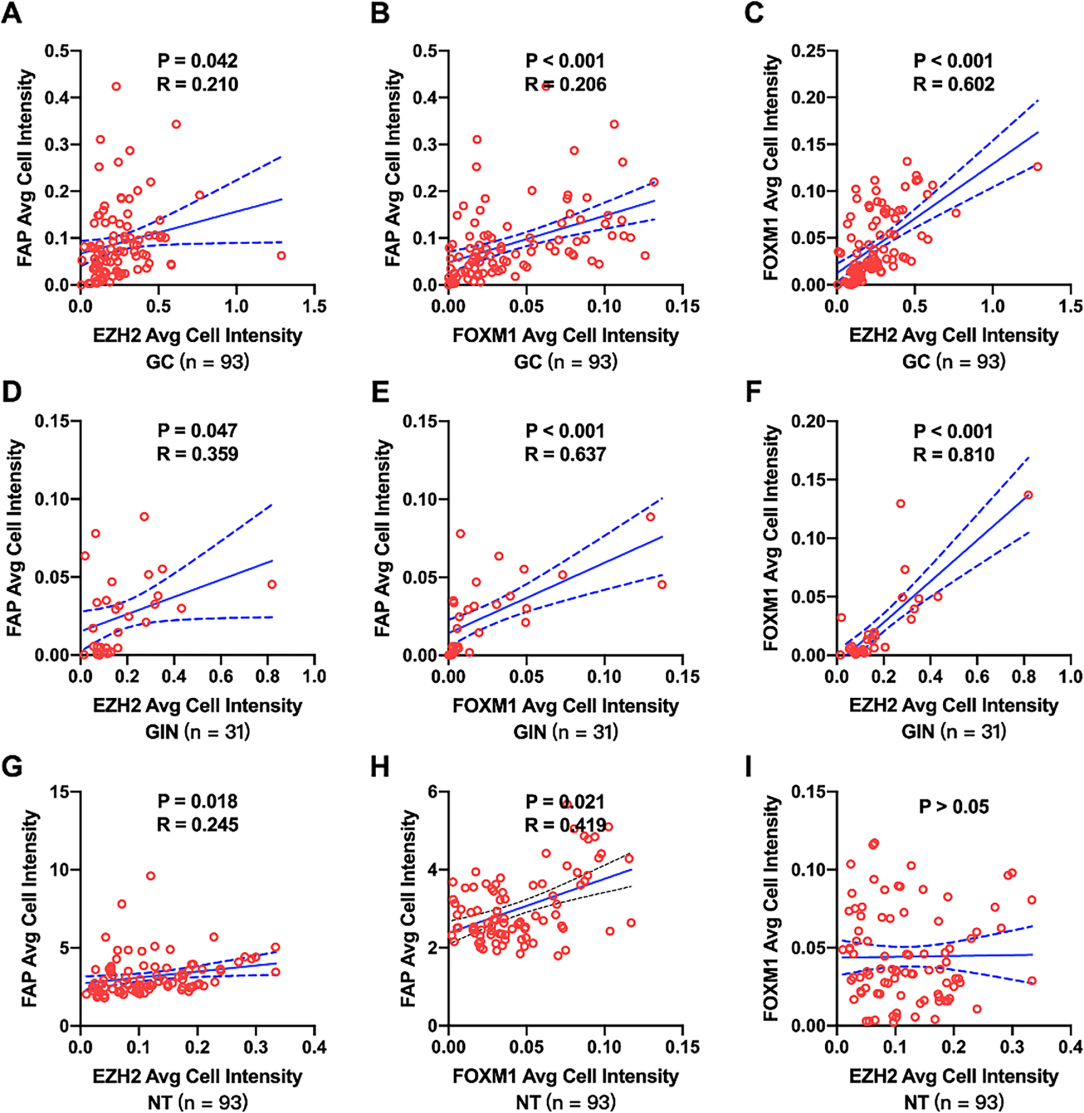
Correlation among EZH2, FOXM1 and FAP expression in GC tissue. The correlation among the average fluorescence intensity of EZH2, FOXM1 and FAP in GC (**A**-**C**), GIN (**D**-**F**) and normal (**G**-**I**) tissue samples. Statistical tests were analyzed using Pearson’s correlation

**Fig. 7:**
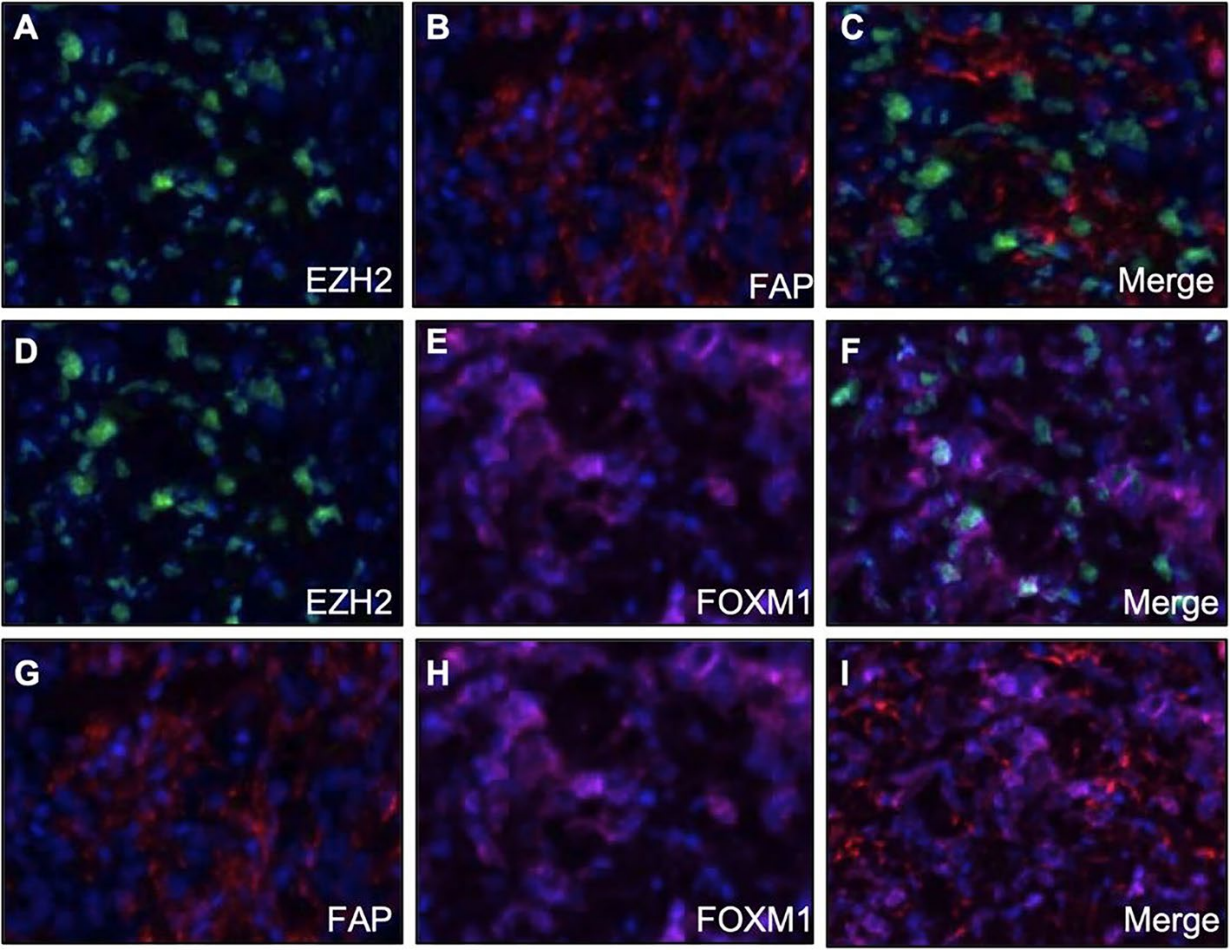
mIF images showed the colocalization of EZH2, FOXM1 and FAP in GC tissue. **A**-**C** EZH2 (**A**) and FAP (**B**) immunoreactivity are colocalized in GC tissues, as seen in the merged image (**C**); **D**-**F** EZH2 (**D**) and FOXM1 (**E**) are colocalized in the merged image (**F**); **G**-**I** FAP (**G**) and FOXM1 (**H**) are also colocalized in GC tissues, as seen in the merged image (**I**)

We then investigated the correlation of the percentage of EZH2, FOXM1 and FAP positive cells in each kind of gastric tissue samples (Fig. 4). There was no significant correlation between the percentage of FAP positive cells with those of EZH2 in GC (Fig. 4 A), GIN (Fig. 4 D) or normal (Fig. 4 G) samples. In GC samples, the percentage of FAP positive cells was positively correlated with those of FOXM1 (r = 0.476, *P* < 0.001, Fig. 4 B), and the percentage of EZH2 positive cells was positively correlated with those of FOXM1 (r = 0.499, *P* < 0.001, Fig. 4 C). In GIN samples, the percentage of FAP positive cells was positively correlated with those of FOXM1 (r = 0.419, *P* = 0.021, Fig. 4 E), and the percentage of EZH2 positive cells was positively correlated with those of FOXM1 (r = 0.565, *P* < 0.001, Fig. 4 F). In normal samples, the percentage of FAP positive cells was positively correlated with those of FOXM1 (r = 0.703, P < 0.001, Fig. 4 H), whereas there was no significant correlation between the percentage of FOXM1 positive cells with those of EZH2 (Fig. 4 I). In addition, the percentage of EZH2 and FOXM1 positive cells was slightly positively correlated with those of FAP in 75 GC cases (r = 0.363, *P* < 0.001, Fig. 5 A), and the percentage of EZH2 or FOXM1 positive cells was positively correlated with those of FAP in 81 GC cases (r = 0.539, *P* < 0.001, Fig. 5 B). Similarly, we also investigated the correlation of the average fluorescence intensity of EZH2, FOXM1 and FAP in each kind of gastric tissue samples (Fig. 6). The average fluorescence intensity of FAP was slightly positively correlated with those of EZH2 in GC (r = 0.210, *P* = 0.042, Fig. 6 A), GIN (r = 0.359, *P* = 0.047, Fig. 6 D) or normal (r = 0.245, *P* = 0.018, Fig. 6 G) samples. The average fluorescence intensity of FAP was slightly positively correlated with that of FOXM1 in GC (r = 0.206, P < 0.001, Fig. 6 B), GIN (r = 0.637, P < 0.001, Fig. 6 E) and normal (r = 0.419, *P* = 0.021, Fig. 6 H) samples. The average fluorescence intensity of EZH2 was significant positively correlated with that of FOXM1 in GC (r = 0.602, P < 0.001, Fig. 6 C) and GIN (r = 0.801, P < 0.001, Fig. 6 F) samples, whereas there was no positive correlation between them in normal samples (Fig. 6 I). Furthermore, we assessed the colocalization of EZH2, FOXM1 and FAP by mIF, which also demonstrated a spatial correlation between EZH2, FOXM1 and FAP positive cells (Fig. 7).

### Correlation between protein expression and clinicopathological factors in GC tissues

Based on the average fluorescence intensity of these three proteins, we divided 93 GC tissue samples into high protein level group and low protein level group according to their Youden value, and then analyzed the correlation between the expression level of these protein and the clinicopathological parameters of GC patients (Table 1). Chi-squared analysis demonstrated that EZH2 protein expression was significantly correlated with tumor location (*P* = 0.005). Moreover, FOXM1 expression were significantly correlated with tumor invasion depth (*P* = 0.030). However, there was no significant correlation between FAP expression and any clinicopathological factors.

**Table 1:** Clinical characteristics of gastric cancer patients

Variables		EZH2 expression		*P* value	FAP expression		P value	FOXM1 expression		P value
		Low (*n* = 14)	High (*n* = 79)		Low (*n* = 51)	High (*n* = 42)		Low (*n* = 59)	High (*n* = 34)	
Age	< 60	8	38	0.533	23	23	0.354	32	14	0.225
	≥60	6	41		28	19		27	20	
Sex	Male	10	65	0.344	41	34	0.946	48	27	0.819
	Female	4	14		10	8		11	7	
Tumor location	Cardia/Proximal	1	16	0.005*	8	9	0.527	13	4	0.547
	Fundus/Body	2	27		14	15		19	10	
	Antrum/Distal	11	23		20	14		20	14	
	Other	0	13		9	4		7	6	
Tumor size	< 5 cm	11	51	0.305	35	27	0.658	40	22	0.761
	≥ 5 cm	3	28		16	15		19	12	
Histologic grade	Poor or undifferentiated	11	58	0.919	40	29	0.527	44	25	0.919
	Good or moderate	3	17		10	10		13	7	
Vascular invasion	Absent	6	33	0.940	20	19	0.558	21	18	0.103
	Present	8	46		31	23		38	16	
Nervous invasion	Absent	8	35	0.375	23	20	0.808	28	15	0.756
	Present	6	44		28	22		31	19	
Tumor invasion depth	T1 + 2	4	15	0.412	11	8	0.764	8	11	0.030^*^
	T3 + 4	10	64		40	34		51	23	
lymphatic metastasis	Absent	3	18	0.911	14	7	0.216	13	8	0.868
	Present	11	61		37	35		46	26	
M stage	M0	10	60	0.718	39	31	0.767	42	28	0.229
	M1	4	19		12	11		17	6	
TNM stage	I + II	4	29	0.558	19	14	0.694	18	15	0.186
	III + IV	10	50		32	28		41	19	

#### Prognostic correlation of EZH2, FOXM1 and FAP in GC tissues

**Fig. 8:**
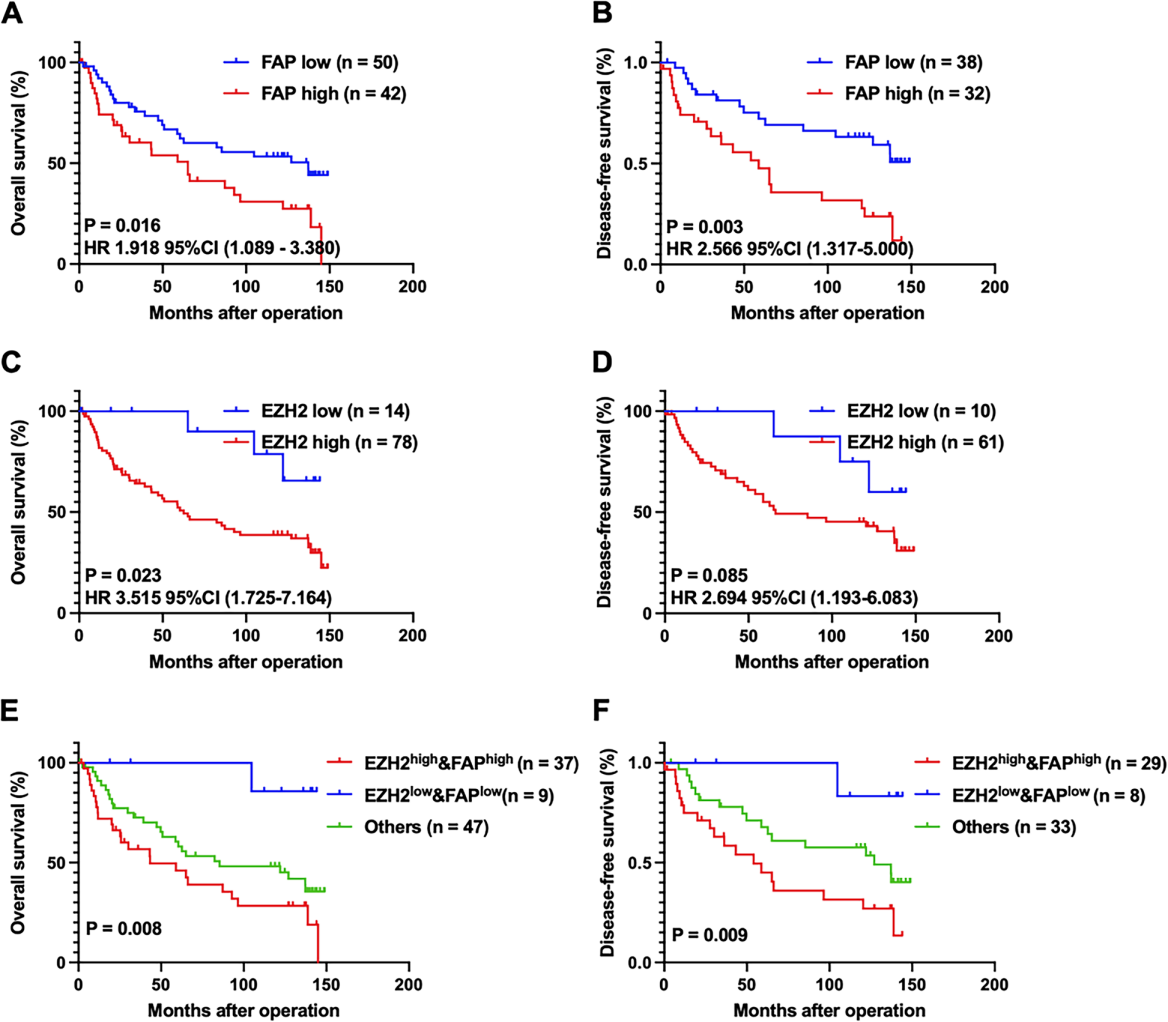
Kaplan-Meier survival curves showed OS and DFS in patients with GC. **A**-**B** The Kaplan–Meier survival curve with log-rank analysis of OS (**A**) and DFS (**B**) showed statistical significance between the curves of patients with FAP high-expression and low-expression, which was classified by the Youden index of the average fluorescence intensity of FAP. **C**-**D** The Kaplan–Meier survival curve with log-rank analysis of OS (**C**) and DFS (**D**) showed statistical significance between curves of patients with EZH2 high-expression and low-expression, which was classified by the Youden index of the average fluorescence intensity of EZH2. **E**-**F** The Kaplan–Meier survival curve with log-rank analysis of OS (**C**) and DFS (**D**) showed statistical significance between curves of patients with indicated protein expression level

Patients with low FAP protein expression exhibited a significantly better OS (*P* = 0.016, Fig. 8 A) and DFS (*P* = 0.003, Fig. 8 B) than those with high FAP protein expression. Analogously, these data also demonstrated that high EZH2 expression conferred a the significantly worse OS in patients with GC (*P* = 0.023, Fig. 8 C). However, patients with higher EZH2 protein expression showed slightly worse DFS than those with lower EZH2 expression (*P* = 0.085, Fig. 8 D); and FOXM1 protein expression showed no prognostication value on OS or DFS (data not shown). In addition, patients with low EZH2 and FAP expression had much better OS (*P* = 0.008) and DFS (*P* = 0.009) than patients with other combinations of EZH2 and FAP expression (Fig. 8 E-F). Our results suggest that the combination of EZH2 and FAP expression may be utilized as powerful factors for prognostication in GC.

Univariate analysis of survival revealed that pTNM stage (*P* = 0.018), peritoneal metastasis (*P* = 0.014), lymphatic metastasis (*P* = 0.004) and vascular invasion (P = 0.008), high expression of FAP (P = 0.018) and EZH2 (*P* = 0.034) were prognostic indicators of OS (Table 2); vascular invasion (P = 0.009), lymphatic metastasis (P = 0.008) and high expression of FAP protein (*P* = 0.006) were prognostic indicators for DFS (Table 3). Multivariate Cox regression analysis showed that high expression of EZH2 (*P* = 0.033) in addition to lymphatic metastasis (P = 0.014) were independent risk factors of OS (Table 2); whereas high FAP protein expression (P = 0.018) in addition to lymphatic metastasis (*P* = 0.017) were independent prognostic indicators for DFS in GC patients (Table 3). In the present cohort, there was no relationship between FOXM1 expression and DFS or OS in patients with GC (Tables 2–3). Overall, these results suggest that EZH2 and FAP are potential independent prognostic factor for GC.

**Table 2:** Univariate and multivariate analyses of clinicopathological factors for overall survival in gastric cancer patients

Variable	Univariate analysis		Multivariate analysis	
	HR (95% CI)	p^a^	HR (95% CI)	p^a^
Age (≥ 60/< 60)	0.896 (0.519-1.548)	0.695		
Gender (Male/Female)	0.984 (0.493-1.963)	0.963		
Location (Cardia/ Fundus/ Antrum/Other)	1.047 (0.773-1.418)	0.767		
Tumor mass size (≥ 5/< 5)	1.739 (0.997-3.031)	0.051		
Histologic grade (Poor, Undifferented /Good, mod)	1.007 (0.512-1.981)	0.984		
Tumor depth (T3, T4/T1, T2)	1.509 (0.733-3.108)	0.264		
Vascular invasion (Present/Absent)	2.268 (1.239-4.152)	**0.008**		
Nervous invasion (Present/Absent)	1.442 (0.825-2.521)	0.198		
Lymphatic metastasis (Present/Absent)	3.867 (1.534-9.748)	**0.004**	3.300 (1.276-8.533)	**0.014**
Peritoneal metastasis (Present/Absent)	2.137 (1.164-3.922)	**0.014**		
pTNM stage (III + IV/I + II)	2.104 (1.137-3.891)	**0.018**		
FOXM1 protein (High/Low)	1.007 (0.581-1.747)	0.980		
FAP protein (High/Low)	1.939 (1.120-3.357)	**0.018**		
EZH2 protein (High/Low)	3.544 (1.102-11.395)	**0.034**	3.584 (1.110-11.570)	**0.033**

*HR* Hazard ratio, *CI* confidence interval; ^a^ All statistical tests were 2-sided. Significance level: p < 0.05

**Table 3:** Univariate and multivariate analyses of clinicopathological factors for disease-free survival in gastric cancer patients

Variable	Univariate analysis		Multivariate analysis	
	HR (95% CI)	p^a^	HR (95% CI)	p^a^
Age (≥ 60/< 60)	0.759 (0.397-1.453)	0.406		
Gender (Male/Female)	0.880 (0.402-1.926)	0.748		
Location (Cardia/Fundus/ Antrum/Other)	1.103 (0.762-1.597)	0.603		
Tumor mass size (≥ 5/< 5)	1.384 (0.694-2.759)	0.356		
Histologic grade (Poor, Undifferented /Good, mod)	1.002 (0.452-2.224)	0.996		
Tumor depth (T3, T4/T1, T2)	1.267 (0.579-2.774)	0.554		
Vascular invasion (Present/Absent)	2.562 (1.262-5.204)	**0.009**		
Nervous invasion (Present/Absent)	1.530 (0.792-2.954)	0.206		
Lymphatic metastasis (Present/Absent)	3.620 (1.406-9.317)	**0.008**	3.205 (1.237-8.305)	**0.017**
pTNM stage (III + IV/I + II)	1.780 (0.914-3.466)	0.090		
FOXM1 protein (High/Low)	1.075 (0.563-2.053)	0.827		
FAP protein (High/Low)	2.534 (1.311-4.899)	**0.006**	2.218 (1.145-4.297)	**0.018**
EZH2 protein (High/Low)	2.646 (0.811-8.625)	0.107		

*HR* Hazard ratio, *CI* confidence interval; ^a^ All statistical tests were 2-sided. Significance level: p < 0.05

## Discussion

In this present study, we firstly aim to investigating the immunostaining of three common CAF markers (FAP, FSP1 and α-SMA) in GC. Consistent with previous study [24], our results also showed that both α-SMA and FSP1 were universally expressed in GC TME, that α-SMA can localized in CAFs, smooth muscle cells and pericytes; FSP1 were stained CAFs, immune cells and epithelial cells. Hereby, α-SMA and FSP1 are not specific markers for CAFs. However, FAP was only specific expressed in the fibroblasts. In addition, we found that FAP expression was significantly higher in tumor CAFs than those fibroblasts in normal tissue. FAP is a prolyl-specific serine proteinase that highly overexpressed in fibroblasts especially at sites of active tissue remodeling [25]. Therefore, we identified FAP as the most specific CAF marker in GC. Correspondingly, the percentage of FAP positive cells can be used to evaluate the proportion of CAFs in GC. Furthermore, FAP protein expression is an independent factor for predicting DFS of patients with GC, which further confirmed the previous reports results [4]. High FAP expression has been proposed as a biomarker for disease progression in metastatic tumor [25]. And FAP plays a seemingly ever-increasing role in tumor development, especially in relation to tumor initiation and metastasis [26]. Thus, FAP^+^CAFs may be functionally associated with the metastatic potential of tumor cells.

The oncogenic role of transcriptional regulator EZH2 has been well confirmed in human malignancies. In the previous study, we reported that EZH2 can transcriptional suppressed PTEN expression and thus promoting tumor stemness and growth by activating AKT signaling pathway [16]. EZH2 was also a transcription co-activator and had been reported to exert its impact by binding and interacting with other transcriptional regulators such as FOXM1 [17]. We also have confirmed FOXM1 as a pivotal onco-protein in GC by binding to lncRNA PVT1 and form a positive regulating loop [27]. In the present study, we simultaneously detected the immunostaining of EZH2 and FOXM1 in gastric samples and confirmed that both EZH2 and FOXM1 were upregulated during GC development once again. Although there was no difference in the expression of FOXM1 between normal tissues and tumor tissues, but its expression in GIN samples was significantly lower than that in GC tissues, suggesting that the expression of FOXM1 is time-specific and its tumor-promoting role in tumors may not be mainly depends on changes in protein expression. Whether FOXM1 achieves its regulatory function through changes in its spatial distribution in GC cells deserves further analysis and identification. In particular, the expression intensity and proportion of EZH2 and FOXM1 showed positive correlation in GC, which may indicate that there is also a potential interaction between the two in GC. However, it seems that EZH2 and FOXM1 are not always strongly expressed in specific GC cell. Moreover, the expression intensity of FOXM1 in cells is generally higher than that of EZH2, whereas EZH2 expression showed predive value for OS in GC. These difference in spatial distribution, expression intensity and prognostication to some extent suggests the difference in mechanism underlying the oncogenic role of EZH2 and FOXM1.

EZH2 in CAFs can participates the peritoneal tumor formation of GC by demethylating H3K27me3 markers [28]; On the other hand, CAFs-derived VEGF promoting the proliferation and angiogenesis of human umbilical vein endothelial cells via regulating EZH2/VASH1 pathway [26]. CAFs can promote cell proliferation through activation of PI3K/Akt signaling in cancer cell [29]. Meanwhile, PI3K/Akt signaling suppresses the expression of FOXO3a, whose inactivation released a switch in promoter occupancy from FOXM1 [30]. Therefore, the PI3K/Akt/FOXO3a axis may provide the link between CAFs and FOXM1 in cancer cell. These studies suggest the potential correlation between EZH2, FOXM1 and CAFs in TME. Thus, we evaluate this possibility using mIF, our spatial and expression analysis results also confirmed this correlation: the CAFs abundance in GC TME, which was represent by the percentage of FAP positive cells, was positively correlated with EZH2 and FOXM1 positive cells. In addition, there were positive spatial correlations among EZH2/FOXM1 positive cells and CAFs. These suggest that tumor cells expressing EZH2 and FOXM1 may be pathological associated with CAFs in GC. The communication mechanism between EZH2 and/or FOXM1-positive GC cells and CAFs needs further analysis. In addition, our results demonstrated that the expression of FAP was positively correlated with EZH2 and FOXM1. Therefore, in TMA of GC, the tumor-promoting effect of FOXM1 and EZH2 in tumor cells may be correlated to the upregulation of FAP in CAFs.

We further determined the clinical significance of EZH2, FOXM1and FAP proteins expression in GC samples. In consistent with our previous observations by immunochemistry, our mIF results also showed that high EZH2 protein expression conferred significantly poorer OS and DFS [16]. The promoting role of FOXM1 in the invasion phenotype of GC cells, which was demonstrated in our previously study [16], also reflect by the positive association between the average fluorescence intensity of FOXM1 and tumor invasion depth. These findings provide support for the critical roles of elevated EZH2 and FOXM1 protein expression in the tumorigenesis of GC.

## Conclusion

In the present study, we evaluated the expression of EZH2, FOXM1 and CAF markers in benign and malignant gastric tissue samples. Both EZH2 and FOXM1 expression was positively associated with CAFs abundance in GC TME. Based on their positive correlation, EZH2 and FOXM1 could be potential cellular target for therapeutic intervention, especially in patients with a large number of CAFs.

## Data Availability

The raw data supporting the conclusions of this article will be made available by the authors, without undue reservation.

## Abbreviations

GC: gastric cancer
mIF: multi-plex immunofluorescence
GIN: gastric intraepithelial neoplasia
OS: overall survival
DFS: disease-free survival
TME: tumor microenvironment
ECM: extracellular matrix
CAFs: cancer-associated fibroblasts
α-SMA: α-smooth muscle actin
FSP1: fibroblast-specific protein 1
FAP: fibroblast activation protein alpha
EZH2: enhancer of zeste homologue 2
FUSCC: Fudan University Shanghai Cancer Center
PRC2: polycomb repressive complex 2
H3K27me: methylation of histone 3 lysine 27
FOXM1: forkhead box M1
TMA: tissue microarray
HR: hazard ratio
CI: confidence interval
